# Modified Pentylenetetrazole Model for Acute Seizure Induction in Rats

**DOI:** 10.3390/biomedicines13112642

**Published:** 2025-10-28

**Authors:** Aseel Saadi, Sereen Sandouka, Rhoda Olowe Taiwo, Yara Sheeni, Tawfeeq Shekh-Ahmad

**Affiliations:** The Institute for Drug Research, The School of Pharmacy, Faculty of Medicine, The Hebrew University of Jerusalem, Jerusalem 91120, Israel; aseel.saadi@mail.huji.ac.il (A.S.); sereen.sandouka@mail.huji.ac.il (S.S.); rhoda.olowe@mail.huji.ac.il (R.O.T.); yara.sheeni@mail.huji.ac.il (Y.S.)

**Keywords:** pentylenetetrazole (PTZ), acute seizure, modified animal model, ECoG, behavioral

## Abstract

**Background/Objectives:** Acute seizure models are widely used in epilepsy research to investigate seizure mechanisms and evaluate antiseizure drug efficacy. Among these models, pentylenetetrazole (PTZ)-induced seizures provide a controlled and reproducible approach to study acute seizure dynamics. However, existing PTZ protocols often suffer from inconsistent seizure induction, high mortality rates, and limited translational relevance. **Methods:** In this study, we systematically evaluated different PTZ dosing regimens in adult Sprague-Dawley rats to establish an optimized acute seizure model that balances seizure induction efficiency with minimal lethality. **Results:** We tested multiple PTZ administration protocols, identifying a two-step regimen of 50 mg/kg followed by 30 mg/kg (30 min later) as the most effective strategy. This dosing approach reliably induced generalized tonic-clonic seizures in 94% of the animals while eliminating mortality. Seizures induced by this regimen were validated through electrocorticography (ECoG) recordings. The behavioral and ECoG assessments of seizures showed a strong agreement in the latency and duration (r = 0.97, *p* < 0.0001; r = 0.81, *p* < 0.05, respectively) with minimal bias in Bland–Altman analysis, confirming that both methods provide statistically comparable and interchangeable measures of seizure characteristics. **Conclusions**: Our findings highlight the robustness and reproducibility of this modified PTZ-induced acute seizure model, offering an improved preclinical platform for studying seizure pathophysiology and screening for novel therapeutic interventions.

## 1. Introduction

Epilepsy is a debilitating neurological disorder characterized by recurrent seizures that affects millions of people worldwide and imposes a significant burden on healthcare systems [1,2,3]. Despite decades of research and development of numerous antiseizure medications (ASMs), a substantial proportion of patients (up to 30%) continue to experience drug-resistant seizures, highlighting the urgent need for novel therapeutic strategies [4,5]. Animal models play a crucial role in epilepsy research by aiding in identifying pathophysiological mechanisms and developing improved treatment options [6,7,8]. However, the currently available models are not without limitations, as they often fail to fully replicate the heterogeneous nature of epilepsy and its diverse seizure phenotypes. While chronic models, such as kindling and genetic rodent models, have been extensively used to study long-term epileptogenesis [9,10,11], these models require prolonged experimental timelines and may not be optimal for studying the acute effects of seizures or for the rapid screening of potential antiseizure therapies [12,13]. Furthermore, chronic models may overlook key biochemical processes associated with early seizure events, as they typically fail to capture the immediate neurophysiological changes occurring at seizure onset. Conversely, acute seizure models, which involve the administration of chemoconvulsants to induce seizures in a controlled and reproducible manner, provide an efficient approach for evaluating antiseizure drug efficacy and investigating seizure pathophysiology [6,11]. One of the most commonly used chemical chemoconvulsants is pentylenetetrazole (PTZ, also termed Metrazol^®^, Cardiazol^®^, or Pentetrazole), which acts as a gamma-aminobutyric acid (GABA) receptor antagonist [14,15]. Since its initial synthesis in 1924, PTZ has been a cornerstone of seizure model development, enabling researchers to study seizure progression, screen for antiseizure compounds, and explore the mechanisms underlying neuronal excitability and seizure initiation [10].

Although PTZ models are widely used for their ability to induce reproducible seizure-like activity in a controlled environment, their translational value remains limited due to the inherent variability in seizure onset and severity, high mortality rates, inconsistent responses among animals, and the need for repeated PTZ administration in kindling protocols to achieve sustained seizure activity (Table 1) [16,17,18,19]. Additionally, although chronic PTZ-induced seizures contribute to the study of epileptogenesis, they do not always capture the acute neurophysiological changes that are crucial for understanding seizure onset and testing fast-acting therapeutic interventions. These limitations have prompted efforts to refine PTZ-based seizure models and optimize dosing regimens to enhance their reproducibility, predictive validity, and applicability in antiseizure drug development.

Here, we systematically evaluated different PTZ dosing schemes in adult Sprague- Dawley (SD) rats to determine their impact on seizure induction and associated outcomes. By comparing the incidence of seizures, seizure severity, and mortality rates across these regimens, we sought to identify a dosing strategy that maximizes seizure induction with minimal mortality. Our results highlight a promising PTZ dosing regimen that may advance research into seizure mechanisms and treatment development.

Although traditional PTZ models remain valuable tools in epilepsy research, they have notable limitations. Our proposed acute seizure rat model provides an efficient approach for studying short-term seizure effects, thereby enhancing our understanding of acute seizure dynamics and paving the way for innovative therapeutic strategies.

## 2. Material and Methods

### 2.1. Animals

Animal experiments adhered to the guidelines set by the Association for Assessment and Accreditation of Laboratory Animal Care International and were approved by the Institutional Animal Care and Use Committee of the Hebrew University of Jerusalem (Approval number: MD-20-16254-5). Male Sprague-Dawley (SD) rats, aged 7–8 weeks and weighing 180–220 g, were used in this study. Rats were obtained from Harlan, Jerusalem, Israel (RRID). A total of 72 animals were used in this study, with an initial allocation of 6–8 animals per experimental group. Rats were individually housed, given free access to food and water, and kept under controlled conditions (12-h light/dark cycle, 23 ± 1 °C, and 50–60% humidity). All animals were acclimated to these conditions for 3–7 days prior to the start of the experiments. No formal statistical power calculation was performed to predetermine the sample size; instead, group sizes were based on prior experience with similar experimental designs and established methodological standards in seizure model studies. Efforts were made to reduce the number of animals used in this study by optimizing experimental design, using within-subject comparisons where possible, and conducting pilot studies to refine dosing regimens before full-scale experimentation. To mitigate suffering, a 5% glucose solution in 0.9% sodium chloride was administered to all animals after seizures, and the animals were closely monitored. Only animals that recovered without any signs of ill health, except for seizures, were included in the study. No animals were excluded from the analysis. However, 6/6 animals in the 100 mg dosing group and 1/6 animals in the 70 + 30 mg group died during the experimental procedures.

### 2.2. Video-ECoG Recording

The animals were acclimated to the housing conditions for at least 7 days before the experiments. Male SD rats were anesthetized with isoflurane and positioned in a stereotaxic frame (Kopf, Tujunga, CA, USA). Prior to surgery, the rats received subcutaneous injections of buprenorphine (0.2 mg/kg) and Metacam (1 mg/kg) for pain management purposes. A subcutaneous electrocorticography (ECoG) transmitter (A3049, Passband: 0.16–160 Hz; Sample rate: 512 SPS; Dynamic range: 30 mV; Open-Source Instruments, Waltham, MA, USA) was implanted. The subdural intracranial recording electrode was placed above the right hippocampus (2.5 mm lateral and 4 mm posterior to the bregma), and a reference electrode was inserted into the contralateral hemisphere (2.5 mm lateral and 6 mm posterior to the bregma). The electrodes were secured to the skull with 1–2 skull screws and tissue glue, followed by dental cement. Immediately after surgery, the rats received subcutaneous injections of 3–5 mL glucose and amoxicillin (Betamox LA, 100 mg/kg, Northwich, Cheshire, England, UK). Postoperatively, the animals were housed individually in cages and closely monitored for pain and distress. As a postoperative analgesic, Metacam (1 mg/kg) was administered subcutaneously once daily for two days. After surgery, the rats were allowed to recover for 7–10 days before starting the experiments. On the day of the experiment, the actual baseline ECoG was recorded for 15–30 min before PTZ injections. Post-PTZ observations lasted for a minimum of 1 h and ended when both behavior and ECoG activity were normalized against the baseline recordings for each individual rat. Data acquisition, control operations, and subsequent analysis were conducted using LWDAQ software, v10.5.5 (Open-Source Instruments). Artifacts in the raw ECoG traces, such as electrical noise, exploratory behavior, and grooming, were manually identified and excluded based on abnormal, non-physiological signal characteristics-such as abrupt high-amplitude deflections, flat lines, or irregular waveform discontinuities-clearly distinct from epileptiform activity. The ECoG epochs corresponding to each behavioral seizure stage after PTZ administration were classified as epileptiform spikes.

### 2.3. Acute Seizure Induction with PTZ and Experimental Groups

**Scheme 1:**
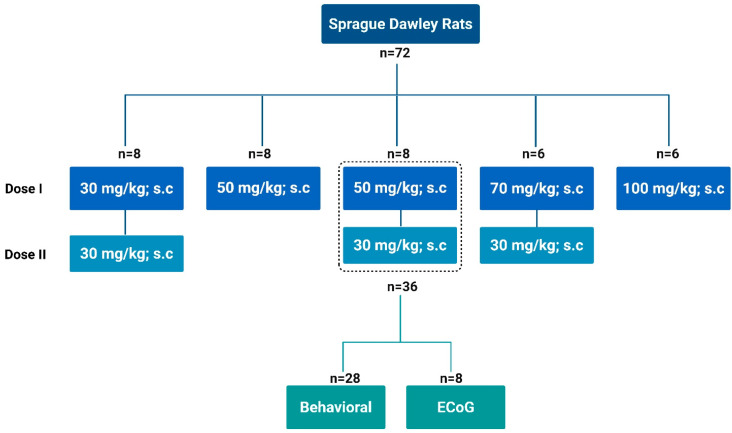
Experimental Design and Dosing Regimen. 36 rats were randomly assigned to receive a single subcutaneous (s.c.) dose of 30, 50, 70, or 100 mg/kg (Dose I, dark blue). A subset of animals in the 30 mg/kg and 50 mg/kg groups (*n* = 8 each) received a second dose of 30 mg/kg s.c. (Dose II, light blue). The group that received50 mg/kg followed by 30 mg/kg (dashed box) showed optimal seizure induction with low mortality. Based on these optimized parameters, this group was expanded for downstream analyses (*n* = 36), including behavioral assessments (*n* = 28) and electrocorticographic (ECoG) recordings (*n* = 8).

We systematically evaluated various subcutaneous PTZ dosing regimens (Sigma-Aldrich^TM^, St. Louis, MO, USA; purity ≥ 99%) for acute seizure induction in –SD rats as illustrated in Scheme 1. PTZ was freshly dissolved in 0.9% saline at a concentration of 50 mg/mL before each experiment, and the solution was used within one hour of preparation. The dosing regimens included a single dose of 100 mg/kg, a combination of 70 mg/kg followed by 30 mg/kg, 30 mg/kg followed by 30 mg/kg, a single 50 mg/kg dose, and a final regimen of 50 mg/kg followed by 30 mg/kg 30 min later. Each dosing protocol was tested in groups of 6–8 rats. Among the regimens, the 50 mg/kg followed by 30 mg/kg regimen was the most effective, resulting in the highest seizure incidence and scores while maintaining the lowest mortality rate. Building on this, we expanded the study to include 36 SD rats using this dosing regimen to assess seizure behavior, severity scores, duration, and mortality rates. A subset of eight rats were implanted with ECoG transmitters to monitor brain electrophysiology using ECoG telemetry, providing a detailed assessment of the effects of this dosing regimen on seizure induction and related parameters. Animal behavior was continuously recorded via video throughout the experimental period.

### 2.4. Seizure Scoring Analysis

To evaluate seizures, both electrographic and behavioral monitoring of the animals were conducted. A seizure was electrographically defined as a high-amplitude, rhythmic discharge with a distinct ECoG trace lasting at least 5 s. Seizures were manually marked by identifying the start and stop positions of each electrographic seizure. The behavioral severity of the electrographically defined seizures was assessed by real-time observations, noting all perceived behaviors and their corresponding timestamps (accurate to 1 s). The experiments were recorded using a video camera for monitoring using digital time-locked CCTV cameras (Microseven) placed outside the cages. The analog video signal was synchronized with the recorded ECoG. During ECoG evaluation, the recorded behaviors were cross-referenced, and the ECoG characteristics were correlated with behavioral expressions. In the case of uncertainty, video footage was reviewed to reassess seizures. Seizure severity was evaluated using a modified Racine scale, extending the traditional six-stage classification [30] to include an additional stage (stage 7) representing seizure-induced death (Supplementary Table S1) [31]. The conventional Racine scale provides a well-established framework for categorizing seizure progression based on behavioral manifestations; however, it does not account for mortality, which is a critical outcome in severe seizure models. The addition of stage 7 allows for a more precise assessment of seizure severity and progression, particularly in cases in which high seizure intensity leads to fatality. This modification enhances the sensitivity of seizure scoring, enabling a more accurate evaluation of therapeutic interventions aimed at mitigating seizure-induced mortality. The updated scale classifies seizure severity as follows: **Stage 1**—sudden behavioral arrest, **Stage 2**—Head nodding, manual automatisms and facial jerking, **Stage 3**—rearing accompanied by myoclonic jerks and unilateral forelimb clonus, **Stage 4**—bilateral forelimb clonic seizures (setting), **Stage 5**—clonic, tonic-clonic followed by generalized convulsions (standing), **Stage 6**—tonic, tonic-clonic convulsions characterized by lateral recumbence, wild jumping, lying on side and followed by generalized convulsions with loss of posture, and **Stage 7**—tonic hindlimb extension accompanied by loss of postural reflexes, progressing to respiratory arrest and death. For seizures exhibiting multiple severity classes during one electrographic event, the most severe behavior determined the seizure severity. In line with previous studies if an electrographic seizure showed no behavioral symptoms, a score of 1 was assigned, indicating the animal was likely in a state of behavioral arrest. Only animals with a score of 5 or above were included in the analysis of duration and latency parameters.

### 2.5. Statistical Analysis

All statistical analyses were performed using GraphPad Prism version 9.3 (GraphPad Software, San Diego, CA, USA). Experimenters remained blinded to group assignments throughout seizure scoring and during the final data analysis. Data were assessed for normality before analysis. Group comparisons involving more than two groups were conducted using ordinary one-way analysis of variance (ANOVA) followed by Tukey’s post hoc test for multiple comparisons. Comparisons between two independent groups were performed using unpaired *t*-tests for normally distributed data or Mann–Whitney U test for non-parametric data. Agreement between the two measurement methods (e.g., behavioral observations and ECoG recordings) was evaluated using the Bland–Altman analysis. For each subject, the difference between the two measurements was plotted against the mean of the two values. The mean bias and 95% limits of agreement (mean difference ± 1.96 × standard deviation of the differences) were calculated. No formal outlier tests were performed, and no data points were excluded from the analysis. Statistical significance was set at *p* < 0.05.

## 3. Results

### 3.1. Impact of the Modified Acute Seizure Model on Seizure Incidence, Severity, Duration, and Mortality Rate Compared to Other Doses

**Figure 1:**
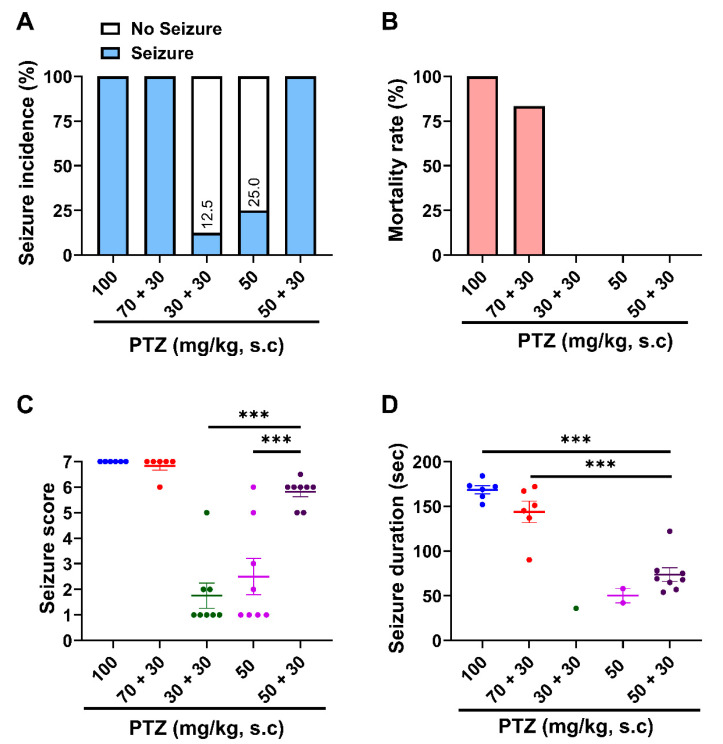
**Evaluation of seizure outcomes across different PTZ dosing schemes.** (**A**) Seizure incidence (%) across various PTZ dosing schemes. (**B**) Mortality rate (%) associated with the PTZ administration schemes in (**A**). (**C**) Seizure severity scores assessed using a modified Racine score (blue = 100 mg/kg; red = 70 + 30 mg/kg; green = 30 + 30 mg/kg; purple = 50 + 30 mg/kg). (**D**) Seizure duration (seconds) across PTZ administration schemes in (**A**) (colors correspond to the same treatment groups as in C). Data are presented as mean ± sem. Statistical significance was determined using Ordinary one-way ANOVA with multiple comparisons followed by Tukey’s post-hoc test. *** denotes *p* < 0.001; (**C**): F (4, 31) = 31.54, *p* < 0.0001; (**D**): F (4, 18) = 27.68, *p* < 0.0001.

To establish an optimal dosing regimen for inducing acute seizures in rats, we tested several protocols for PTZ administration. Although a single 100 mg/kg PTZ dose proved uniformly lethal (Figure 1A–C), pilot the split-dose regimen of 70 mg/kg followed by 30 mg/kg still induced severe (stage 7) seizures, resulting in rapid mortality of 5 out of 6 animals within 2–3 min post-seizure (Figure 1A–C). The first dose was subsequently reduced to 30 mg/kg, followed by a second 30 mg/kg injection 30 min later. This modification successfully eliminated mortality but resulted in seizures in only 12.5% (1/8) of the animals that exhibited generalized seizures of stage 5 or above (Figure 1A,B). Similarly, a single subcutaneous dose of 50 mg/kg resulted in seizures in only 25% (2/8) of rats, with no mortality observed (Figure 1A,B).

Based on these findings, we tested a modified two-step regimen (50 mg/kg + 30 mg/kg) to balance seizure induction and mortality risks. This approach successfully induced seizures in all animals (8/8) while eliminating lethality (Figure 1A,B). The severity and duration of seizures were assessed for each regimen (Figure 1C,D). The high-dose protocols (100 mg/kg and 70 + 30 mg/kg) induced stage 6–7 seizures lasting approximately 3 min, ultimately leading to death. In contrast, the 30 + 30 mg/kg and single-dose 50 mg/kg regimens resulted in a maximum seizure score of 5–6, characterized by head nodding, excessive rearing, and forelimb clonus.

The most effective protocol was the two-dose regimen of 50 mg/kg, followed by 30 mg/kg 30 min later. This approach consistently induced stage 6 seizures, characterized by clonic and tonic-clonic convulsions, including lateral recumbency and bilateral forelimb clonus. In this group, all rats exhibited seizures that lasted up to 1 min (Figure 1C,D).

### 3.2. Seizure Incidence and Characteristics Following Optimal Dose Selection

**Figure 2:**
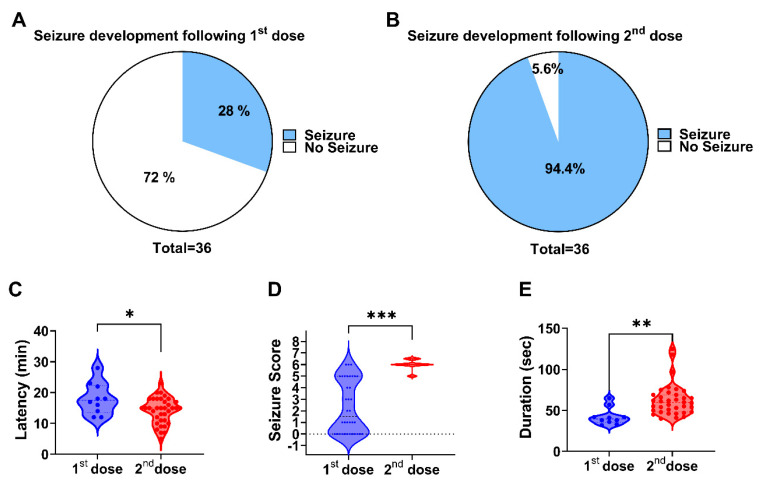
**Seizure incidence and characteristics following the first and second administration of the selected dosing regimen.** (**A**,**B**) Proportion of rats exhibiting seizures after the first (**A**) and second (**B**) injection. (**C**) Seizure latency (time to seizure onset) following the first and second doses (**D**) Seizure severity scores following the first (blue) and second doses (red) (**E**) Seizure duration (seconds) after each dose. Data are presented as mean ± sem. Statistical significance was assessed using an unpaired *t*-test (**C**,**E**) and Mann-Whitney test (**D**). (**C**): *p* = 0.0118; (**D**): *p* < 0.0001; (**E**): *p* = 0.0011. * denotes *p* < 0.05; ** denotes *p* < 0.01; *** denotes *p* < 0.001.

After identifying the most effective PTZ dosing regimen (50 mg/kg followed by 30 mg/kg 30 min later), we conducted a detailed evaluation of the seizure incidence and characteristics. Specifically, we assessed the percentage of animals exhibiting seizures after each dose, along with the seizure severity (score), duration, and latency. Thirty-six rats were included in this experiment, of which 8 rats were subjected to ECoG recording. Our findings indicate that after the first PTZ injection, only 28% of the rats (10/36) exhibited seizures of stage 5 or above, with an average seizure score of 2.3 (Figure 2A,D). Given this limited response, a second dose was necessary to achieve robust seizure induction. Following the second PTZ injection, seizure incidence increased markedly, with 94% of the rats exhibiting stage 5–7 seizures, with an average seizure score of 5.9 (Figure 2B,D). The latency to seizure onset after the first and second injections was approximately 19 min and 14 min, respectively (Figure 2C). The average seizure (stage 5–7) duration following the first and second doses was 43 and 63 s, respectively (Figure 2E). These results confirmed the efficacy of the 50 mg/kg + 30 mg/kg PTZ regimen in reliably inducing seizures while maintaining controlled and measurable responses.

### 3.3. ECoG Signature of Seizures Following PTZ

**Figure 3:**
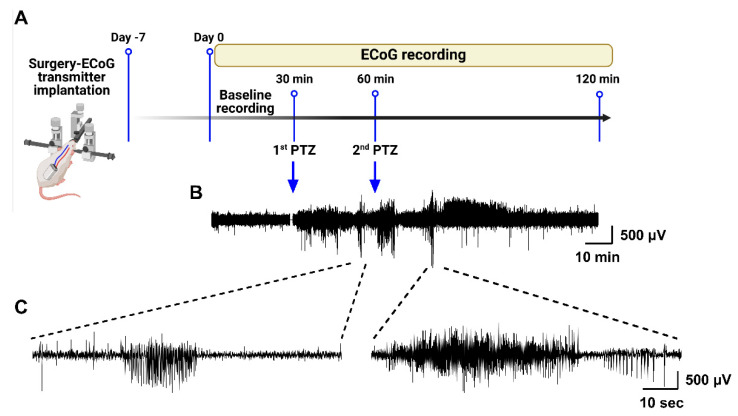
**Experimental timeline and representative ECoG traces of PTZ-induced seizure activity.** (**A**) Schematic representation of the experimental timeline, illustrating the sequence of surgical intervention followed by PTZ administration and continuous ECoG monitoring. (**B**) Representative ECoG traces capturing a 2-h ECoG recording post-PTZ administration, demonstrating dynamic seizure activity. (**C**) Zoomed-in segment of the ECoG trace in (**B**), illustrating seizure-specific high-amplitude, high-frequency discharges, characteristic of PTZ-induced ictal activity.

To confirm seizure characteristics, we performed ECoG recordings in 8/36 rats implanted with telemetry transmitters (Open-Source Instruments) and subjected to the most effective PTZ dosing regimen (50 mg/kg followed by 30 mg/kg 30 min later). The experimental timeline is shown in Figure 3A. Following a 30-min baseline ECoG recording, a subcutaneous injection of 50 mg/kg PTZ was administered, followed by a second dose of PTZ (30 mg/kg) 30 min later. ECoG recordings revealed the onset of brief seizure activity approximately 15 min post-injection (Figure 3B). Generalized tonic-clonic seizures were observed 15–20 min after the second injection (Supplementary Video S1). This sequential dosing regimen enabled the identification of distinct ECoG seizure signatures corresponding to the first and second PTZ-induced seizures.

**Figure 4:**
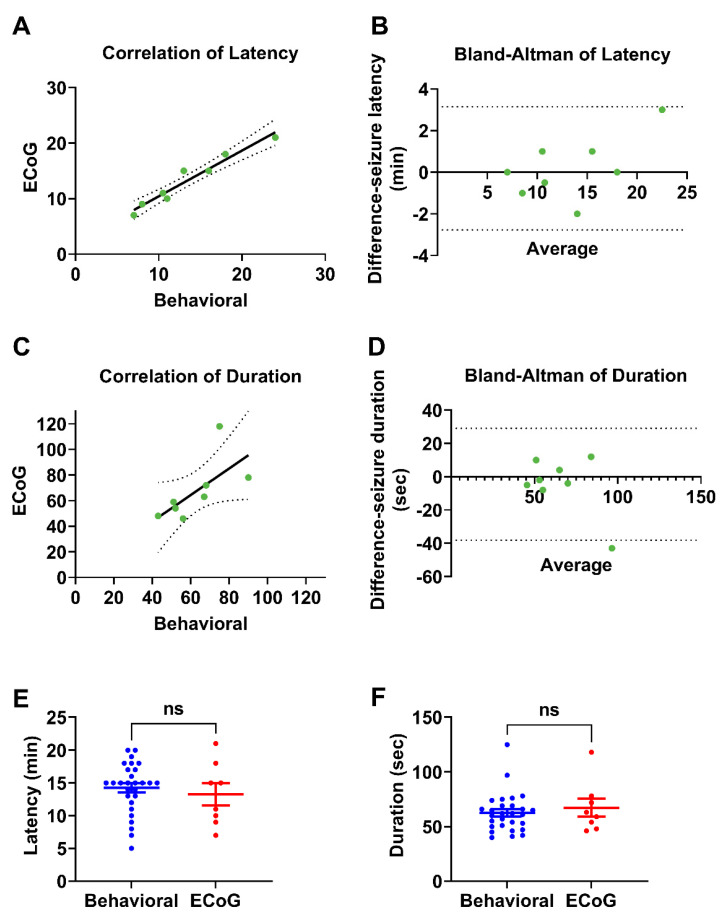
**Comparison of seizure onset latency and duration assessed by behavioral observation and ECoG recordings.** (**A**) Correlation analysis of seizure onset latency measured using behavioral scoring and electrocorticography (ECoG). (**B**) Bland–Altman plot comparing seizure onset latency measurements from behavioral and ECoG methods. The y-axis shows the difference between the two methods (Behavior-ECoG), and the x-axis represents the mean of the two measurements. The solid line denotes the mean bias, and dashed lines indicate the 95% limits of agreement. (**C**) Correlation of seizure duration between behavioral and ECoG assessments. (**D**) Bland–Altman plot for seizure duration measurements between the two modalities. Each dot represents data from a single animal; solid line indicates best fit, and the dotted lines denote the 95% confidence interval. (**E**,**F**) Direct comparison of seizure onset latency (**E**) and seizure duration (**F**) between two independent experimental cohorts: (1) animals evaluated using continuous ECoG recordings (*n* = 8); and (2) animals assessed exclusively via behavioral observation (*n* = 28). ns = not significant.

To further validate the seizure metrics, ECoG data from the implanted rats were compared with the behavioral seizure assessments conducted in same animals, and further with the remaining 28 animals (without ECoG data). The agreement between ECoG and behavioral methods for assessing latency to seizure and seizure duration was evaluated using Pearson correlation and Bland–Altman analysis. A strong positive correlation was observed between the two methods for assessing seizure latency (r = 0.97, *p* < 0.0001), indicating a high degree of linear association (Figure 4A). Bland-Altman analysis showed a mean bias of 0.188 (min), with 95% limits of agreement ranging from −2.77 to +3.15 (Figure 4B). Similarly, a positive correlation was observed in the assessment of seizure duration (r = 0.81, *p* < 0.05; Figure 4C). The Bland-Altman analysis showed a mean bias of 4.5 (s), with 95% limits of agreement ranging from −38.1 to +29.1 (Figure 4D). The narrow limits of agreement and negligible mean difference confirm that both methods yield statistically comparable results and can be used interchangeably to assess seizure characteristics.

To confirm the consistency of our findings across different assessment modalities, we compared two independent cohorts of animals: (1) rats evaluated using continuous electrocorticography (ECoG; *n* = 8) and (2) rats assessed exclusively through behavioral monitoring (*n* = 28). A direct comparison between these groups revealed no statistically significant differences in seizure onset latency and total seizure duration (Figure 4E,F). These results indicate a high degree of concordance between electrophysiological and behavioral evaluations, supporting the reliability of behavioral assessments in the absence of ECoG recordings.

## 4. Discussion

In the present study, we systematically evaluated various PTZ dosing regimens to establish an optimized model for acute seizure induction in SD rats. This strain was chosen given their widespread use in acute PTZ-induced seizure paradigms, their well-characterized baseline seizure susceptibility, and their suitability for stereotaxic implantation of ECoG electrodes. Importantly, strain-specific differences in seizure thresholds and responses to convulsants have been documented. For ref. [32] reported that Wistar and Long-Evans rats may display altered latencies to seizure onset and different susceptibility profiles compared toSD. These findings underscore the possibility that seizure incidence and severity may vary across strains. While our study focused on SD to ensure methodological consistency with prior literature, future investigations incorporating additional strains and female cohorts will be essential to determine the broader generalizability of this modified PTZ model.

Our findings demonstrated that a two-dose regimen of 50 mg/kg followed by 30 mg/kg 30 min later was the most effective at inducing seizures with minimal mortality. This protocol reliably produced generalized tonic-clonic seizures (stage 6) while maintaining a controlled and measurable response. Importantly, our study highlights the robustness of behavioral seizure assessments by validating them against ECoG recordings, which exhibit a strong concordance between electrophysiological and observational measures of seizure duration and latency.

PTZ is a widely used convulsant for studying epilepsy and screening antiseizure drugs [33,34]; however, conventional dosing regimens present challenges (Table 1), including high mortality rates, inconsistent seizure induction, and prolonged administration protocols that lead to dynamic changes in seizure states and anxiety-like behaviors [23].

In this study, we systematically optimized PTZ dosing to develop a reliable and ethically improved acute seizure model in SD. Initial doses were guided by prior literature [23,33] and refined empirically through stepwise testing of single and two-step regimens (30 + 30 mg/kg, 50 mg/kg, 70 + 30 mg/kg, 100 mg/kg). This approach balances effective seizure induction with animal welfare, and the strong concordance between behavioral scoring and ECoG recordings validates the reliability of this refined model for preclinical epilepsy research. Our results revealed that a single high dose (100 mg/kg) consistently induced seizures but led to an unacceptably high mortality rate. In contrast, split-dose regimens with lower cumulative doses (e.g., 30 + 30 mg/kg) significantly reduced mortality but failed to induce reliable seizure activity. The 50 + 30 mg/kg regimen emerged as the optimal compromise, ensuring a high seizure incidence (94%) without any lethal outcomes. Our findings establish a refined PTZ-induced acute seizure model with improved reproducibility and reduced mortality, addressing a critical limitation of the conventional protocols. This approach enhances the utility of PTZ in preclinical epilepsy research, particularly for high-throughput screening of antiseizure drugs.

The effectiveness of this regimen may be attributed to the pharmacokinetics of PTZ and its effects on neuronal excitability [35,36]. The first dose (50 mg/kg) likely initiates subthreshold neuronal hyperexcitability, priming the brain for a more robust seizure response upon the administration of the second dose (30 mg/kg). This two-step approach may mimic the conditions of heightened seizure susceptibility seen in certain forms of epilepsy, making it a useful model for studying acute seizure mechanisms and potential interventions.

A key finding of this study was that behavioral seizure manifestations closely aligned with the electrophysiological data recorded via ECoG. Our comparison between the ECoG-monitored rats (*n* = 8) and behaviorally observed rats (*n* = 28) revealed no significant differences in seizure latency and duration. These results validate the use of behavioral scoring in acute PTZ models, reinforcing its utility in preclinical epilepsy research where ECoG monitoring is not always feasible. The high level of agreement between the two assessment methods enhances confidence in the reliability of the behavioral seizure characterization.

Although ECoG recordings provide crucial insights into seizure dynamics, they require specialized equipment and surgical implantation of transmitters, which may not be feasible in all experimental settings. Our findings support the notion that detailed behavioral seizure scoring remains a valuable tool for assessing seizure activity in preclinical studies, particularly when high-throughput approaches are required to assess seizure activity. However, future studies should further refine behavioral scoring methods by integrating automated video tracking and machine-learning algorithms to enhance precision and reduce observer bias.

The establishment of an optimized PTZ dosing regimen has significant implications for preclinical epilepsy research. The 50 + 30 mg/kg protocol provides a reproducible, high-throughput method for studying seizure mechanisms while minimizing experimental variability. This is particularly advantageous for investigating the pathophysiological processes underlying seizure initiation and propagation and for testing novel antiseizure drugs. Furthermore, by refining acute seizure induction, this model bridges the gap between chronic epilepsy models and drug screening approaches, offering a faster and more ethically sound alternative to repeated PTZ administration (i.e., kindling).

The ability to reliably induce seizures with a controlled and measurable response facilitates the exploration of seizure-induced molecular and cellular alterations. For example, this model can be used to assess changes in oxidative stress markers, inflammatory pathways, and synaptic plasticity following acute seizures. These insights can inform the development of targeted interventions aimed at mitigating seizure-related neurotoxicity and at reducing the risk of epilepsy in susceptible individuals.

Although our study successfully identified an optimal PTZ dosing strategy, several limitations should be acknowledged. First, the long-term consequences of acute PTZ-induced seizures were not examined, and further research is needed to determine whether this model triggers neuroplastic changes associated with the development of epilepsy. Second, while the optimized PTZ regimen offers a robust tool for acute seizure studies, acute PTZ-induced seizures cannot recapitulate the chronic, heterogeneous, and multifactorial pathophysiology of human epilepsy. Additionally, although behavioral scoring and ECoG data were highly correlated, future studies should incorporate multimodal assessments, including EEG power spectral analysis and histological markers of neuronal damage, to validate this model further.

Furthermore, in this study we restricted our experiments to male SD rats to minimize biological variability and establish a standardized acute seizure model. Previous work has demonstrated that sex hormones influence seizure thresholds and pharmacological responses [37,38,39], and including both sexes would have introduced additional complexity beyond the scope of the present study. Our primary objective was to optimize a reproducible PTZ dosing regimen with minimal mortality. A key limitation of this study is the use of only male Sprague-Dawley rats; future studies should include both sexes to evaluate sex-specific variations in seizure susceptibility and treatment response, as supported by accumulating evidence of sexual dimorphism in experimental and clinical epilepsy research. Nonetheless, future studies should systematically examine sex-specific differences in seizure susceptibility and antiseizure drug efficacy using this refined model.

Another important limitation is the exclusive use of Sprague–Dawley rats. This strain was selected due to its widespread adoption in PTZ-induced seizure paradigms, its well-characterized baseline seizure susceptibility, and its suitability for stereotaxic ECoG implantation, thereby ensuring methodological consistency with prior work. Nevertheless, accumulating evidence demonstrates pronounced strain-dependent variability in both seizure thresholds and PTZ pharmacokinetics [32,40,41]. For instance, Wistar and Long–Evans rats exhibit distinct latencies to seizure onset and divergent susceptibility profiles compared with SD rats. Such inter strain differences highlight that seizure incidence, severity, and even pharmacological responses cannot be assumed to generalize across rodent populations. Future studies that extend this optimized protocol to additional strains, genetically epilepsy-prone models, and female cohorts will be critical to establish its robustness, enhance translational validity, and capture the biological heterogeneity that more closely reflects the complexity of human epilepsy.

## 5. Conclusions

This study establishes a refined, highly reproducible PTZ-induced acute seizure model in Sprague Dawley rats, employing a two-step 50 + 30 mg/kg subcutaneous protocol. This regimen reliably induces generalized stage 6 seizures in 94% of animals while effectively eliminating mortality, representing a significant improvement over conventional high-dose or single-step protocols. Validation of behavioral seizure scoring against ECoG recordings confirmed strong concordance, reinforcing the reliability of observational assessments in preclinical epilepsy research.

The novelty of this model lies in its ability to combine robust seizure induction with controlled and measurable outcomes within a short, sub-hour timeline, facilitating high-throughput studies. This approach offers a robust platform for screening candidate antiseizure compounds, as well as investigating undiscovered mechanisms underlying seizure emergence and propagation. Future studies should include different species of different strains and both sexes to better assess the biological variability, incorporate automated seizure detection paradigms to minimize observer bias, and to examine behavioral, biochemical and histological changes after seizures. These refinements are expected to strengthen the translational relevance and mechanistic insight of the optimized PTZ model, contributing to the advancement of new therapeutic approaches for epilepsy.

## Figures and Tables

**Table 1:** Summary of PTZ-Induced Seizure Models in Various Animal Species.

Model	Comments	Animal Species	Dose
Kindled Seizure Models	Chronic PTZ administration can alter its metabolism and lead to tolerance, which may skew long-term study results by affecting seizure thresholds and responses [20] Exhibit variable seizure thresholds and behaviors [21] Dynamic alterations in seizure activity and anxiety-related behaviors [9] The kindling process requires repeated PTZ administrations over extended periods, making it time-consuming and costly [6]	Swiss mice	Repeated 35 mg/kg, i.p., every other day for 15 days [20]
		Beagle dogs	15 mg/kg, i.v. infusion [22]
		Long-Evans rats	Repeated 40 mg/kg, i.p., every other day for 20 days [23]
		Wistar rats	First dose of PTZ 35 mg/kg, i.p., every alternate day [24]
		Zebrafish	Repeated 80 mg/kg, for 10 days [25]
		Sprague-Dawley Rats	80 mg/kg of PTZ, i.p., for 30 days [26]
Acute Seizure Models	High doses of PTZ cause neurotoxicity, leading to death [21] Not fully represent the range of seizure types [27] High variability in seizure onset, duration, and severity depending on dose, age and species [27]	Swiss mice, C57BL/6J	80 mg/kg of PTZ, i.p. [19,28]
		Swiss mice, C57BL/6J	40–65 mg/kg, i.p. [27]
		Wistar rats	35 mg/kg, i.p. [19]
		Zebrafish	125–500 mg/kg [29]

## Data Availability

The original contributions presented in this study are included in the article. Further inquiries can be directed to the corresponding author.

## Supplementary Materials

The following supporting information can be downloaded at: https://www.mdpi.com/article/10.3390/biomedicines13112642/s1, Table S1: Modified Racine Scale for Behavioral Seizure Scoring; Video S1: Representative Video of Behavioral Seizure Activity.
