# Supplementary material for: Modified Pentylenetetrazole Model for Acute Seizure Induction in Rats
Source: Biomedicines. 2025 Oct 28;13(11):2642. doi: 10.3390/biomedicines13112642 (PMC12650221; doi:10.3390/biomedicines13112642)
Supplement: Supplementary file 1 [file biomedicines-13-02642-s001.zip › biomedicines-3908709-Supplementary.pdf]

Supplementary Material

Modified Pentylenetetrazole Model for Acute Seizure Induction in Rats

Aseel Saadi, Sereen Sandouka, Rhoda Olowe Taiwo, Yara Sheeni and Tawfeeq Shekh-Ahmad *

Supplementary Table S1. Modified Racine Scale for Behavioural Seizure Scoring

Severity Score	Behavioral Description	Seizure Phenotype Visualization
**1**	Sudden behavioral arrest	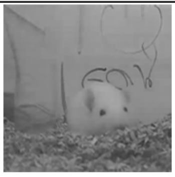
**2**	Head nodding, manual automatisms and facial jerking	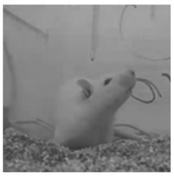
**3**	Myoclonic jerks, unilateral forelimb clonus	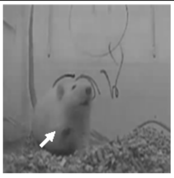
**4**	Bilateral forelimb clonus (setting)	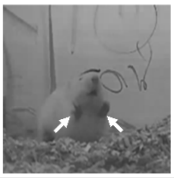
**5**	Clonic, tonic-clonic followed by generalized convulsions (standing)	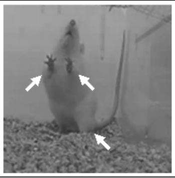
**6**	Tonic, Tonic-clonic convulsions with loss of posture	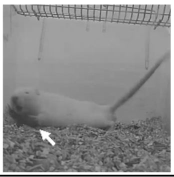
**7**	Tonic extension, respiratory arrest, death	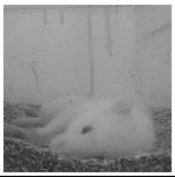

Summary table that quantifies the severity scores corresponding to each stage intensity in addition to a representative figure depicting the characteristic behavioral manifestations observed at each seizure stage, facilitating a standardized classification of seizure (Supplementary table 1). This visual representation enhances the qualitative understanding of seizure severity, demonstrating the distinct motor phenotypes associated with each stage, from initial facial movements to generalized tonic-clonic seizures and seizure-induced death. The incorporation of stage 7 in the modified scale enables a more precise differentiation between non-fatal and fatal seizures, allowing for improved assessment of seizure progression and therapeutic intervention efficacy.

Supplementary Video S1. Representative Video of Behavioral Seizure Activity

Rat experienced stage 6 of generalized tonic-clonic seizure that was confirmed by video recording approximately 15- 20 minutes following the second dose – for 78 seconds.
